# How to change workflow to enhance implementation of professional services in community pharmacies: A deprescribing case study

**DOI:** 10.1177/17151635241246975

**Published:** 2024-05-06

**Authors:** Kelda Newport, Aili V. Langford, Aisling M. McEvoy, Deborah V. Kelly, Tara Smith, Cara Tannenbaum, Justin P. Turner

**Affiliations:** School of Pharmacy, Memorial University of Newfoundland, Newfoundland and Labrador; Centre for Medicines Use and Safety, Faculty of Pharmacy and Pharmaceutical Sciences, Monash University, Victoria, Australia; Centre for Medicines Use and Safety, Faculty of Pharmacy and Pharmaceutical Sciences, Monash University, Victoria, Australia; School of Pharmacy, Memorial University of Newfoundland, Newfoundland and Labrador; School of Pharmacy, Memorial University of Newfoundland, Newfoundland and Labrador; Centre de recherche, Institut universitaire de gériatrie de Montréal, Québec; Faculté de Médicine, Université de Montréal, Québec; Centre for Medicines Use and Safety, Faculty of Pharmacy and Pharmaceutical Sciences, Monash University, Victoria, Australia; Centre de recherche, Institut universitaire de gériatrie de Montréal, Québec; Faculté de Pharmacie, Université de Montréal, Québec; Faculté de Pharmacie, Laval Université, Québec

## Introduction

Canadian community pharmacies dispensed more than 758 million prescriptions in 2020,^1^ supporting Canadians in treating, managing and preventing disease. Despite their many benefits, medications carry potential risks and may be inappropriate for an individual if more is taken than needed, if taken for longer than necessary, or when the potential for harm outweighs the potential for benefit.^2^ Potentially inappropriate medications are prescribed at a high volume across Canada, with Newfoundland and Labrador (NL) having some of the highest prevalence.^3,4^ Amid growing awareness of the harms of potentially inappropriate medication use, “deprescribing” (the supervised process of withdrawing an inappropriate medication) has been identified as a mechanism to engage health care providers and patients in a shared decision-making process to improve appropriate medication use.^5^

Canadian research has demonstrated that pharmacist-led deprescribing can reduce the use of potentially inappropriate medications.^6^ Rolling out this professional practice model nationally has the potential to reduce patient harm and generate substantial financial savings.^7,8^ In 2019, working with the Pharmacists’ Association of Newfoundland and Labrador, the NL government implemented a pharmacist-led, patient-centred, interprofessional service in community pharmacies to reduce the potentially inappropriate use of proton pump inhibitors (PPIs) and sedative-hypnotics (benzodiazepines and z-drugs) in community-dwelling adults.

Much of the research to date on deprescribing in community pharmacies is derived from randomized controlled trials. Although many trials have been successful, there are little data to guide the implementation of these evidence-based practices into everyday workflow. The Consolidated Framework for Implementation Research highlights the need to consider contexts to successfully implement evidence-based interventions, including settings outside the pharmacy, within the pharmacy and individual staff roles and characteristics.^9^ This article describes activities and processes that facilitated implementation of deprescribing in community pharmacies and offers practical suggestions for pharmacists to incorporate professional services into their pharmacy.^10^

## Methods

### Description of intervention

SaferMedsNL (www.SaferMedsNL.ca) was a theory-driven, evidence-based, province-wide initiative to promote safe medication use by deprescribing in NL. Two successful evidence-based interventions to promote deprescribing were adapted and implemented across the province: a public awareness campaign (comprising TV, radio and social media advertising) coupled with health care provider education and direct patient education by pharmacists, family doctors and nurse practitioners.^6,11^

### Study design and sample

Multidisciplinary focus groups were conducted to investigate the uptake of deprescribing professional services across NL. Full methods have been described elsewhere^12^; however, in brief, pharmacy students, pharmacists, nurse practitioners and physicians were invited to participate in focus groups to understand their experiences with implementing deprescribing in practice. The focus of this article is on identifying enablers to implementing the professional service, deprescribing, into the community pharmacy workflow by analyzing the responses of practising community pharmacists and third-year pharmacy students from Memorial University. Ethics for this research was approved by the NL Human Research Ethics Board (reference Nos. 2019.085 and 2020.040).

A purposive sampling approach was undertaken, and 2 sets of focus groups were conducted. In 2019, third-year pharmacy students completing community pharmacy placements as part of their Structured Practice Experience (SPE) III course were recruited to the first focus group. In 2020, practising health care providers were recruited to the second set of focus groups through advertisements distributed via professional associations and social media to include maximum variation across provider types, practice settings and geographical locations. Nominal group technique (NGT) was conducted with each group of participants to allow participants to generate their own ideas while also allowing participants to hear and understand the perspectives of the collective group,^13,14^ in relation to the following question: “*What actions or processes support the implementation of deprescribing into the daily workflow of your practice*?” This analysis focuses on responses from pharmacists and pharmacy students only.

### Data collection

Each focus group lasted approximately 2 hours, led by an experienced facilitator (J.P.T.). Each NGT group was audio recorded or video recorded using Zoom, with audio files transcribed verbatim. Field notes were made by members of the research team. Data from all focus groups were pooled prior to analysis.

### Analysis

Transcripts and field notes were analyzed to identify specific actions and processes that supported the professional service of deprescribing, which were then mapped to key steps within community pharmacy workflow.^15^

## Results

### Participants

Sixteen participants (11 pharmacists, 5 pharmacy students) were recruited. Demographic data pertaining to the specific pharmacy were not collected due to the small number of participants and participating pharmacies in the SPE program. The 11 pharmacists included 7 owners (5 independent, 2 chain) and 4 staff pharmacists (chain pharmacies).

### Factors that support deprescribing

**Figure 1:**
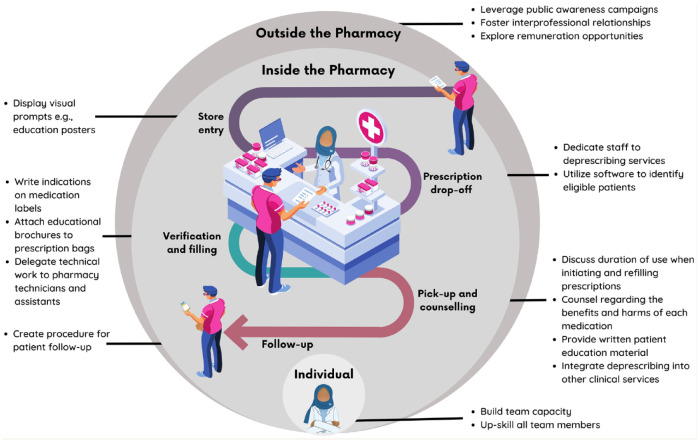
Facilitators to deprescribing in the pharmacy workflow

Factors supporting the implementation of deprescribing professional services in community pharmacy were identified outside the pharmacy, inside the pharmacy and at the individual staff-member level (Figure 1). Practical examples of strategies used by participants at each of these levels are provided in Table 1.

**Table 1:** Actions that support implementation of deprescribing interventions into community pharmacy practice

Action to support the implementation of the professional service	Examples provided by participants
Outside the pharmacy	
Leverage public awareness campaigns	Pharmacies built their own campaigns to encourage patients to speak to their health care provider about their medications, mirroring the province-wide public awareness campaign. Multiple avenues were utilized, such as including social media.
Foster interprofessional relationships	Positive interprofessional relationships were built through networking and community seminars, providing a better understanding of the roles of a pharmacist and how pharmacists, doctors and nurse practitioners can collaboratively improve medication appropriateness. A universal deprescribing plan with predetermined steps was developed with local doctors and nurse practitioners, including preferred communication methods. Pharmacists used the new professional service as an opportunity to introduce themselves to the doctors and nurse practitioners in their local area and discuss their knowledge and expectations of the service.
Explore remuneration opportunities	Remuneration of services has the potential to support the hiring of more pharmacists, facilitating professional services.
Inside the pharmacy	
Pharmacy entry and prescription drop-off	
Display visual prompts	Posters were displayed at the prescription pick-up and drop-off areas to encourage patients to talk to the pharmacist if they were taking any of the medications eligible for deprescribing. Brochures that included information about deprescribing and long-term effects of eligible medications were displayed, to prompt patients to ask if they were eligible for deprescribing. Postcards invited patients to make a booking with the pharmacist to review their medications.
Dedicate staff to deprescribing services	Staff members were trained to be responsible for championing, organizing and quality improvement of the deprescribing service within the pharmacy.
Utilize software to identify eligible patients	Patients eligible for deprescribing were identified: • proactively by printing a list of patients taking PPIs or sedative-hypnotics, • proactively using pharmacy management software or limiting by parameters such as recent refills and • reactively using pop-ups for patient identification and deprescribing reminders.
Prescription verification and filling	
Write indications for medications on the prescription label	Recording the indication for each medication helped to ensure that medications were being used appropriately for the patient’s condition and informed the deprescribing plan. Flagging when further assessment was required.
Attach educational brochures to prescription bags	Attaching reminders, such as the patient educational brochure to the prescription bag, acted as a catalyst for conversations about deprescribing and providing education to patients about the benefits and harms of their medications. Deprescribing forms, booklets, paper clips and other visual reminders were attached to the prescription bag to prompt the pharmacy team to call the pharmacist for consult during pick-up.
Prescription pick-up and counselling	
Discuss intended duration of use when initiating and refilling prescriptions	Discussions about intended duration of use when first dispensing a medication were suggested to create an expectation to deprescribe in the future, improving patient acceptance of the professional service.
Counsel regarding the benefits and harms of each medication	Mentioning potential adverse effects of medications such as PPIs and sedative-hypnotics laid the groundwork for future deprescribing conversations.
Provide written patient education material	Materials such as deprescribing clinical guidelines and algorithms were made accessible to allow pharmacists to use these tools to explain the reasons behind deprescribing and the process and to suggest safer alternatives. Patient handouts introduced the concept of deprescribing to patients in an easy-to-read, patient-friendly language. Providing handouts at this point gave patients time to contemplate their medications, deprescribing and speaking with their pharmacist prior to their next visit.
Integrate deprescribing into other clinical services	Deprescribing was incorporated into other clinical services such as medication reviews.
Follow-up	
Create a procedure for patient follow-up	Electronic calendars or binder call-back systems were used to follow-up patients. Using pharmacy management software follow-up functions, pharmacists could keep track of patients who completed an initial and follow-up deprescribing consult. Postcards with an “appointment date and time” were used to schedule patient appointments for follow-up professional services.
Individual	
Build team capacity	Identify resources and prioritize tasks to support deprescribing and follow-up. Utilize the skills of pharmacy students to improve the deprescribing process.
Up-skill all team members	Provide education and training on deprescribing to all pharmacy staff. Most times, patients’ contact with the pharmacy is through assistants or technicians, which makes it important for them to know about deprescribing and be able to describe it to patients. Provide opportunities for pharmacy students to observe deprescribing counselling. Include deprescribing activities into pharmacy students’ practice experiences.
Delegate technical work to pharmacy assistants	Faxing, billing, scanning and organizing follow-up calendars were delegated to pharmacy assistants and technicians to free up time for pharmacists to engage with patients in deprescribing professional services.

The tools described in this table, including the pharmacist-to-doctor communication tool, patient educational brochures, posters and appointment postcards, are all freely available at www.safermedsnl.ca/resources. PPIs, proton pump inhibitors.

#### Outside the pharmacy

The province-wide public awareness campaign was thought to have prompted patient-initiated deprescribing conversations about the harms and benefits of their medications. Participants described conducting their own health promotion campaigns to complement the province-wide promotion. Participants felt that establishing positive, interprofessional relationships with other members of the multidisciplinary team and developing a deprescribing plan in collaboration with local prescribers also enabled successful deprescribing.

#### Inside the pharmacy

Participants identified a range of actions and activities within the pharmacy that enabled deprescribing. SaferMedsNL resources (such as posters, postcards, videos and patient educational brochures available at www.safermedsnl.ca/resources) were used to varying extents to meet the needs, contexts and preferences of each pharmacy and their patients. Sample strategies were described at multiple workflow stages, including using physical resources at the pharmacy entrance, prescription drop-off, verification and filling, pick-up and counselling and during follow-up (Figure 1 and Table 1).

##### Pharmacy entrance and prescription drop-off

Participants noted opportunities for promoting deprescribing by displaying promotional posters at store entry and intentionally focused use of posters and brochures at the prescription drop-off counter. Staff could use these materials to prompt patients that the pharmacist may want to talk to them about their medication, thus setting the patients’ expectations about the delivery of the professional service.

##### Verification and filling

Within the dispensary, several strategies were identified to support the professional service. Strategies either identified patients or promoted pharmacists. Pop-ups added to the dispensing software were effectively utilized to identify, track, monitor and follow up patients who were eligible, interested in or engaging in deprescribing. Some participants noted this as a reactive prompt (they responded to an alert during the dispensing process), while others described employing proactive processes, setting aside dedicated time each week to look at their dispensing histories and identify eligible patients. Other visual prompts were used to flag patients during the pick-up and counselling stage, including placing the medication in a paper bag and stapling a reminder note to the bag. This was seen as a particularly useful strategy if the patient was coming back to collect their medication.

##### Pick-up and counselling

Participants described providing written education material to patients when the patient picked up their medication. Using standardized tools allowed participants to guide the patient through conversations about the benefits and harms of their medication and discussions about intended durations of use. For example, SaferMedsNL adapted and standardized evidence-based tools for pharmacists to guide the deprescribing discussion (available at www.safermedsnl.ca/resources). Furthermore, it was noted that having a structured patient counselling process provided an opportunity to integrate other professional services such as medication reviews.

##### Follow-up

Creating effective processes for tracking patient trajectories and following up with them was seen as a critical step for ensuring the follow-up professional service was conducted. Participants use a range of strategies including online calendars, dispensing software notes or paper-based binders. Ensuring that the paperwork required for follow-up funding was available along with the calendar reminder facilitated funding for this service.

##### Staff roles and characteristics

Building team capacity through adequate staffing and upskilling of all team members was considered vital. For example, pharmacy assistants were trained to identify eligible medication classes and use the posters at the prescription drop-off to alert patients that the pharmacist might want to talk to them. Similarly, while dispensing a prescription, registered pharmacy technicians could respond to the software alerts by placing a reminder note in the tray along with the prescription for the pharmacist to check. Likewise, delegating other processes to technicians, such as organizing the follow-up reminder system, freed up time, allowing pharmacists to deliver the professional service. Time management and the organizational skills of staff members were considered instrumental in the successful implementation of the professional service. Recognizing and using the skills of pharmacy students was considered important, particularly in busy primary care settings.

## Discussion

Despite legislation an enabling expanded scope of practice for pharmacists in many Canadian provinces, the uptake of patient care services across Canada has been low.^16^ This study identifies and summarizes concrete actions that pharmacists can take to support the implementation of professional services, such as deprescribing, within the community pharmacy setting. Although barriers to implementing deprescribing in practice are well known,^13,17^ we identified practical strategies, both internal and external to the pharmacy, that can enable the integration of evidence-based professional services into pharmacists’ daily workflow.

Participants identified synergism of implementation approaches that targeted both outside and inside the pharmacy. Province-wide public health campaigns, coupled with promotion of evidence-based posters and brochures in the pharmacy, were deemed complementary and reinforced the same message via multiple avenues. Although many factors in the outer setting (e.g., pharmacy remuneration models) are outside the control of individual pharmacy staff members, factors within the inner setting of the pharmacy are able to be successfully changed, with intervention components adapted to suit the needs of end users. Identifying which patients are likely to accept deprescribing can be difficult^18^; therefore, applying strategies to key stages of the pharmacy workflow, including queue time, prescription preparation, stock retrieval, prescription processing, verification, counselling and checkout, can support offering the service to all eligible patients.^15^ Participants embedded strategies to promote the professional service at each workflow stage. Significant variability in activities performed by different participants was identified, with no one pharmacy implementing all strategies. Contextual differences between sites, influenced by factors such as geographic location, patient demographics and workload and staffing pressures, likely resulted in different strategies being more effective in different settings. With contextual differences between pharmacies, it is likely that there is no “one-size-fits-all” approach. Our findings suggest that offering a suite of interventions and implementation strategies that are adaptable to suit specific contexts and workflows is required. Core components of evidence-based interventions such as the public awareness campaign and educational resources should be maintained; however, an adaptable periphery such as changing at what point(s) in the workflow an intervention is embedded, may allow for more acceptable and implementable interventions.^19^

Activities outside the pharmacy (e.g., public health campaigns and public engagement^20^) can have a far reach; nevertheless, our findings suggest that behavioural nudges embedded within the workflow can produce significant impact. For example, behavioural nudges, including posters, pamphlets and audit and feedback to doctors, have demonstrated efficacy in improving antibiotic prescribing.^21,22^ Regarding targeting professional services to address the use of PPIs and sedative-hypnotics, participants described the value of using features within pharmacy software to identify eligible patients and prompt discussions about deprescribing. Previous literature has shown that health care professionals are often reluctant to initiate conversations about deprescribing due to fears of disrupting the patient-provider relationship.^23,24^ However, it has been demonstrated that initiating deprescribing conversations with patients does not harm patient trust.^25^ Therefore, small behavioural nudges that prompt patients and pharmacists to engage in conversations about deprescribing may act as a catalyst for shared decision-making and optimization of medication use. Supported by an organizational culture that values the skills and abilities of all team members and positive external relationships with other health care professionals, behavioural nudges have the capacity to support safe and effective deprescribing in practice.

In conclusion, this study identified several synergistic actions and processes that enabled pharmacists to implement a professional service focused on deprescribing into their daily practice. By considering pharmacy workflow and varying contexts, these findings may be applicable and translatable to implementing patient care services more broadly across Canada. ■
